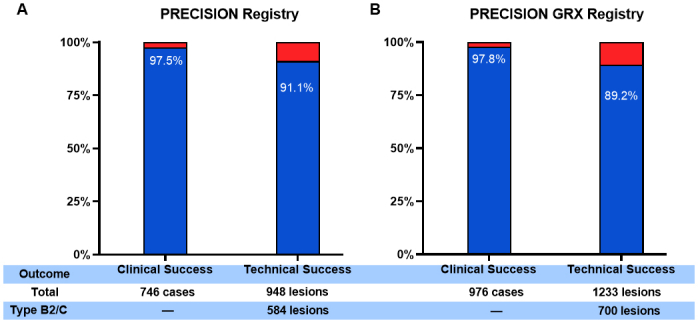# Robotic-Assisted Percutaneous Coronary Intervention: Final Results of the PRECISION and PRECISION GRX Studies

**DOI:** 10.1016/j.jscai.2025.103655

**Published:** 2025-05-13

**Authors:** Ehtisham Mahmud, Ryan D. Madder, David H. Wohns, Jeffrey M. Schussler, Adam Salisbury, Paul Campbell, Tejas M. Patel, William L. Lombardi, William J. Nicholson, Manish A. Parikh, Natia Kelm, Ron Waksman, Alexandra J. Lansky, Giora Weisz

**Affiliations:** a Division of Cardiovascular Medicine, Sulpizio Cardiovascular Center at UC San Diego Health, La Jolla, California; b Division of Cardiology, Frederik Meijer Heart & Vascular Institute, Spectrum Health, Grand Rapids, Michigan; c Division of Cardiology, Baylor University Medical Center, Dallas, Texas; d Division of Cardiology, Saint Luke's Mid America Heart Institute/UMKC, Kansas City, Missouri; e Division of Cardiology, Sanger Heart & Vascular Institute, Carolinas HealthCare System NE, Concord, North Carolina; f Division of Cardiology, Apex Heart Institute, Ahmedabad, India; g Division of Cardiology, University of Washington, Seattle, Washington; h Division of Cardiology, Emory University School of Medicine, Atlanta, Georgia; i Division of Cardiology, NewYork-Presbyterian Brooklyn Methodist Hospital, Weill Cornell Medical School, Brooklyn, New York; j Siemens Healthineers, Newton, Massachusetts; k Division of Cardiology, MedStar Washington Hospital Center, Washington, DC; l Division of Cardiovascular Medicione, Department of Internal Medicine, Yale School of Medicine, New Haven, Connecticut; m Division of Cardiology, NewYork-Presbyterian Hospital/Columbia University Irving Medical Center, New York, New York

**Keywords:** complex coronary lesions, percutaneous coronary intervention, PRECISION, PRECISION GRX, robotic percutaneous coronary intervention

## Abstract

**Background:**

Robotic percutaneous coronary intervention (R-PCI) reduces occupational hazards for interventional cardiologists. However, there is a lack of clinical data in a large patient cohort. The aims of this study were to evaluate the safety and efficacy of R-PCI with both the first (CorPath 200) and second (CorPath GRX) (Corindus, Siemens Company) generation robotic systems.

**Methods:**

These prospective, multicenter, single-armed studies enrolled patients with symptomatic coronary artery disease from 2013-2017 (PRECISION; CorPath 200) and 2017-2020 (PRECISION GRX; CorPath GRX). The primary outcome measures were clinical success, defined as <30% residual stenosis in the absence of major adverse cardiovascular events, and technical success, defined as clinical success without conversion to manual PCI.

**Results:**

A total of 1734 R-PCI procedures (PRECISION: 754 procedures, 950 lesions; PRECISION GRX: 980 procedures, 1233 lesions) were performed. Clinical (96.9% and 98.1% PRECISION and PRECISION GRX respectively, *P* = ns) and technical (89.6% and 89.2% PRECISION and PRECISION GRX respectively, *P* = ns) success rates were similar. Higher clinical success rates were observed in moderate/severe calcification lesions, bifurcation lesions, and long lesions with the second-generation system, and higher technical success rates were observed with the second-generation system in moderate/severe calcification lesions, bifurcation lesions, and angulated lesions.

**Conclusions:**

This multicenter experience with R-PCI demonstrates high clinical and technical success for patients treated with either the first- or second-generation robotic platform. Clinical and technical success rates with the second-generation robotic platform were higher for several complex lesion types. These data support the safety and efficacy of R-PCI in clinical practice.

## Introduction

**Figure 1:**
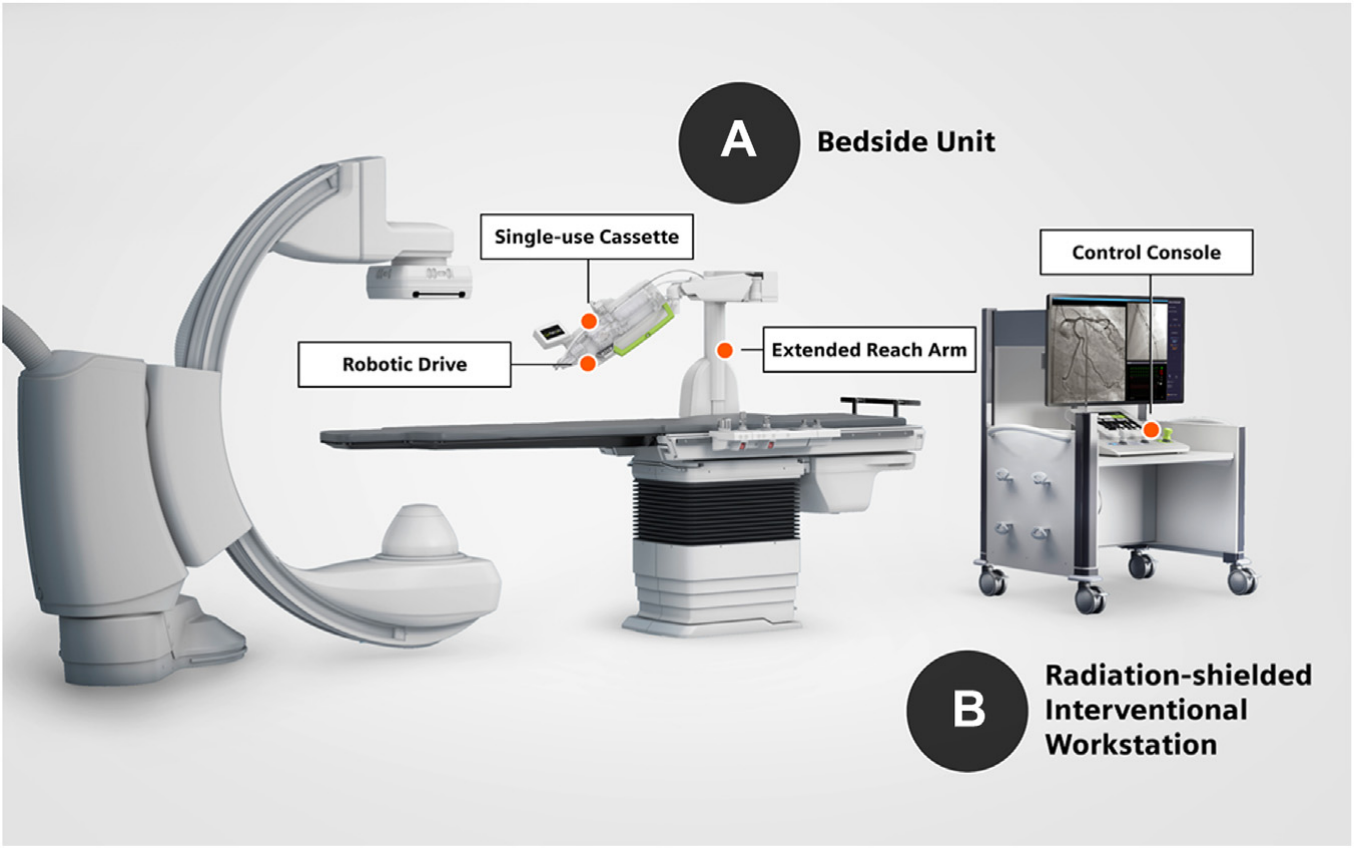
**Overview of the CorPath GRX system in the catheterization laboratory**. (**A**) Tableside unit: consists of an extended reach arm, robotic drive, and single-use cassette which is mounted to the catheterization table and designed to be easily positioned for all access points. (**B**) Radiation-shielded interventional workstation: houses the control console and monitor(s) to display angiographic and hemodynamic data. The interventional workstation can be located in the control room or an optional cockpit.

The approach to percutaneous coronary intervention (PCI) has continued to evolve over the past 4 decades, with PCI now being the primary revascularization strategy for the majority of patients.^1^ Although the procedure has undergone refinement, the associated hazards to the interventional operator and team have not received the same level of attention.^2^ Robotic PCI (R-PCI) enables the remote advancement and manipulation of intracoronary devices by an operator seated behind a shielded interventional cockpit with the goal of addressing both the orthopedic- and radiation-associated risks of PCI. The Percutaneous Robotically Enhanced Coronary Intervention trial, which evaluated the first-generation CorPath 200 Robotic System (Corindus, A Siemens Healthineers Company), reported a clinical success rate of 97.6% in 164 patients with predominantly simple coronary lesions and a reduction of radiation exposure to the operator by 95.2%.^3^ The second-generation CorPath GRX Robotic System provides additional functionality, including active robotic guide catheter control (Figure 1).^4^

Although there have been single-center experiences reported with both the first- and second-generation robotic platforms in the treatment of simple and complex coronary lesions,^4^^,^^5^ questions persist regarding the utility of R-PCI for the treatment of coronary disease encountered in clinical practice. In the present study, we present the first comprehensive report from the PRECISION (A Post-Market Registry for the Evaluation of the CorPath 200 System Effectiveness in Percutaneous Coronary Interventions) and PRECISION GRX (A Multicenter Post-Market Registry for the Evaluation of the CorPath GRX System Effectiveness in Percutaneous Coronary Interventions) registries which were each undertaken as multicenter, multioperator studies to evaluate the safety and efficacy of R-PCI.

## Methods

The PRECISION multicenter registry (NCT01917682) was designed to evaluate the safety, effectiveness, and utility of CorPath 200, and the PRECISION GRX multicenter registry (NCT03278301) was designed to evaluate the safety, effectiveness, and utility of the next-generation CorPath GRX for R-PCI. The intention of both registries was to evaluate outcomes by multiple operators at different clinical centers in a real-world population of patients with coronary artery disease, with a clinical indication for PCI and amenable for R-PCI. The study was funded by Corindus, manufacturer of the robotic system used in both registries. The principal investigators E.M. (PRECISION GRX) and G.W. (PRECISION) drafted the manuscript with review by all participating authors, and verified the authenticity of the data presented.

Both PRECISION and PRECISION GRX studies were approved by the institutional review boards at each participating center. Standardized definitions and case report forms were used for data collection for each of the 2 registry studies. All subjects provided written informed consent for data collection. Outcomes were investigator-reported, and data were monitored with outcomes confirmed. The robotic platforms have been described previously^4^^,^^5^ and are briefly reviewed below.

### Procedural description

#### Robotic platforms for R-PCI systems

The CorPath 200 Robotic System used in the PRECISION study was the first-generation commercially approved system and consists of a radiation-shielded interventional cockpit and tableside unit. The latter includes a robotic articulating arm, which contains the robotic drive. The robotic drive houses a single-use cassette in which 2 separate intracardiac devices (guidewire and coronary balloon/stent delivery system) can be inserted (Central Illustration). After a guide catheter is manually engaged in the coronary artery, the cassette is connected to the guide catheter. The robotic arm is connected by cables to the interventional cockpit. The cockpit includes high-definition monitors displaying fluoroscopic images and hemodynamic data. Touchscreen controls on the console and 2 joysticks allow the operator to robotically advance, manipulate, and retract 0.014-inch guidewires and rapid-exchange balloon/stent catheters. The operator can control fluoroscopy and cineangiography using pedals in the cockpit. The interventional team tableside handles contrast injection and equipment exchanges.

**Central Illustration:**
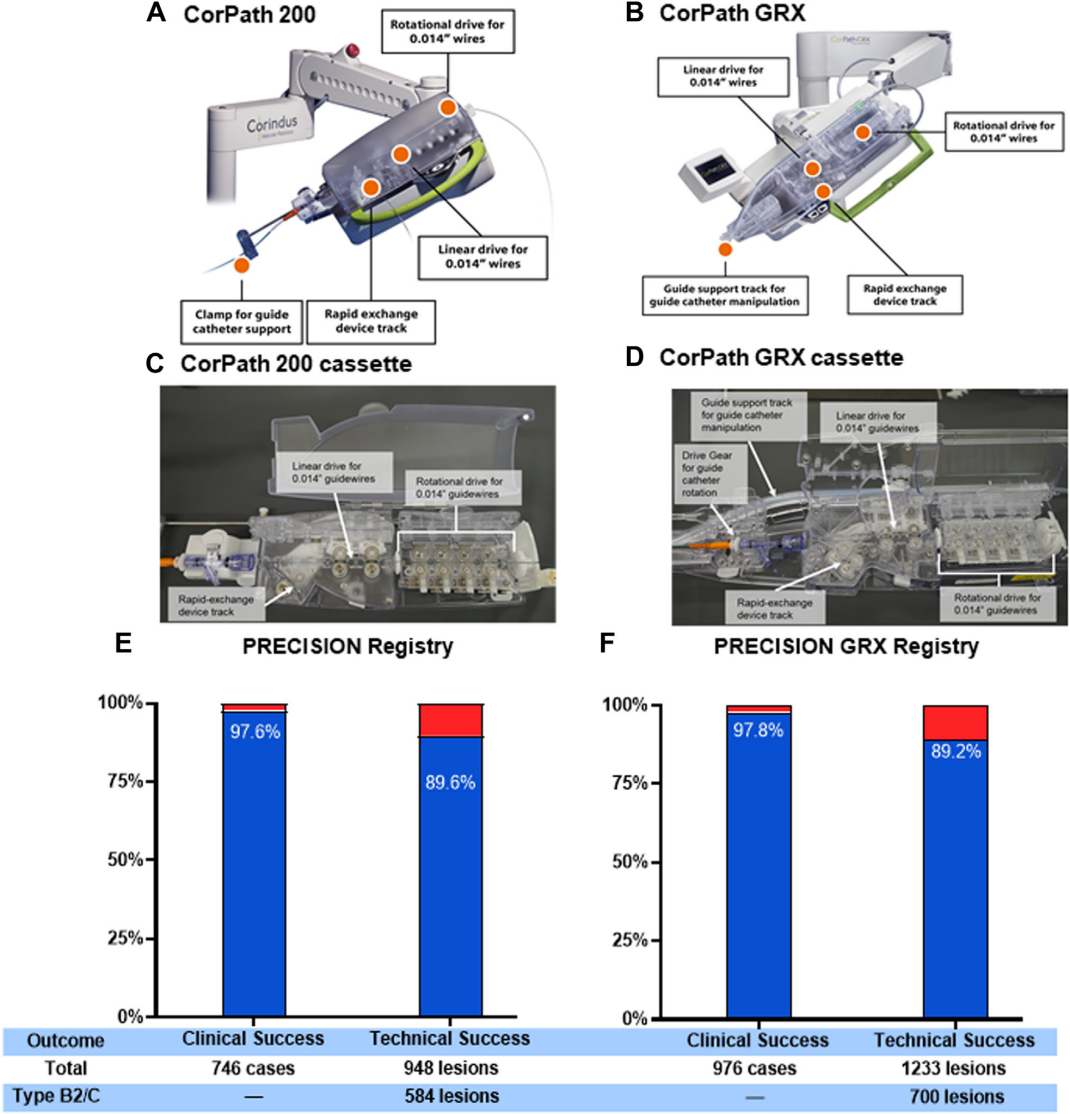
**Robotic platforms with clinical and technical success of the PRECISION and PRECISION GRX registries.** Clinical success (by case) is defined as less than 30% residual stenosis (visual estimate) post percutaneous coronary intervention (PCI) in the lesion(s) treated with the robotic system, without in-hospital major adverse cardiovascular event (MACE). Technical success (by lesion) utilizing the PRECISION GRX definition∗: successful completion of the robotic-assisted PCI without unplanned manual conversion. (A) First-generation robotic CorPath 200 robotic arm. (B) Second-generation robotic CorPath GRX robotic arm. (C) First-generation robotic CorPath 200 single-use cassette. (D) Second-generation robotic CorPath GRX single-use cassette. (E) PRECISION registry: clinical success 97.6% (728/746); technical success 89.6% (850/948), (F) PRECISION GRX registry: clinical success 97.8% (955/976); technical success 89.2% (1100/1233). ∗PRECISION definition: successful completion of robotic-assisted PCI without planned or unplanned manual conversion, see Supplemental Figure S1.

The CorPath GRX, used in the PRECISION GRX study, is the second-generation robotic system offering enhancements to the platform from the first-generation system, by adding key upgrades that increase precision, improve workflow, and extend the capabilities and range of procedures that can be performed robotically. Of note, the robotic drive and single-use cassette have been expanded to include robotic guide catheter control, controlled by a third joystick that has been added to the control console (Figure 1). Additional features of the CorPath GRX System include automated wire movements (technIQ Smart Procedural Automation, Rotate-on-Retract) to aid in guidewire navigation, side-branch access, lesion crossing, and device manipulation.

### Study population

The present study analyzes data from 2 prospective postmarket registries: PRECISION and PRECISION GRX. A total of 31 centers participated: 16 centers participated in PRECISION and 20 centers participated in PRECISION GRX. Three centers were located outside of the United States, and 5 centers enrolled patients for both registries. Procedures were performed from September 2013 to January 2017 for PRECISION and August 2017 to February 2020 for PRECISION GRX.

PRECISION was a multicenter, prospective, single-arm registry collecting data on the safety and effectiveness of the first-generation CorPath 200 Robotic System in PCI procedures. Similarly, PRECISION GRX was a multicenter, prospective, single-arm registry collecting data on the safety and effectiveness of the second-generation CorPath GRX Robotic System used to facilitate PCI. Inclusion criteria for the registries were the same: age ≥18 years, clinical indication for PCI, and deemed appropriate for R-PCI by the operating physician.

### Definitions (end points)

In the PRECISION registry, clinical success was defined as <30% residual stenosis and final thrombolysis in myocardial infarction flow grade 3 by visual estimate post-PCI in the lesion(s) treated with CorPath 200, without an in-hospital major adverse cardiovascular event (MACE). In-hospital MACE was defined as occurring within 72 hours of the procedure or prior to hospital discharge, whichever occurred first. The definition of MACE included cardiac death, clinically relevant myocardial infarction (MI) after revascularization (Q-wave or non–Q-wave MI),^6^ or clinically driven target vessel revascularization by PCI or coronary artery bypass grafting. Technical success was defined as the proportion of procedures with clinical success in the absence of conversion to manual PCI (M-PCI).

In the PRECISION GRX registry, clinical success was defined as <30% residual stenosis and final thrombolysis in myocardial infarction flow grade 3 by visual estimate post-PCI in the lesion(s) treated with CorPath GRX, without in-hospital MACE. In-hospital MACE was defined as occurring within 72 hours of the procedure or prior to hospital discharge, whichever occurred first. The definition of MACE included cardiac death, clinically relevant MI after revascularization (Q-wave or non–Q-wave MI), or clinically driven target vessel revascularization by PCI or coronary artery bypass grafting. Technical success was defined as the successful completion of robotic-assisted PCI of the target lesion(s) without unplanned conversion to M-PCI owing to the inability of the guidewire or balloon stent/catheter to navigate the vessel anatomy or poor guide catheter support.

### Statistical analyses

For the comparison of continuous variables between the studies, the Mann-Whitney *U* test was used. A comparison of categorical variables between the 2 groups was performed using the Fisher exact test. The descriptive summaries of continuous variables were expressed as mean ± SD, and median (IQR) as relevant. Categorical data were expressed in percentage terms with the number of events in the category (n) over the total number of subjects in the analysis population (N). A *P* value of <.05 was considered statistically significant, although all testing was done in an exploratory manner (the study was not powered for testing). Statistical analysis was carried out using SAS version 9.4 (SAS Institute).

## Results

Subject demographic and baseline characteristics are summarized in Table 1. In both registries, a total of 1734 procedures (PRECISION, 754; PRECISION GRX, 980) with 2183 robotically treated lesions (PRECISION, 950; PRECISION GRX, 1233) were included. Seven hundred fifty-four procedures (66.7 ± 12.1 years; 73.50% male subjects) were performed in subjects who underwent R-PCI with the CorPath 200 Robotic System in the PRECISION registry. Nine hundred eighty procedures (65.4 ± 11.6 years; 73.50% male subjects) were performed in subjects treated with the CorPath GRX Robotic System in the PRECISION GRX registry. Baseline characteristics were typical of patients undergoing PCI, with a high frequency of hypertension, diabetes mellitus, and prior history of coronary revascularization (Table 1). Subjects with abnormal stress tests and both acute and chronic coronary syndromes were comparably treated in both registries.

**Table 1:** Baseline characteristics of the study cohorts.

Characteristic	PRECISION (N = 754)	PRECISION GRX (N = 980)	*P* value
Age, y	66.7 ± 12.1	65.4 ± 11.6	.04
Male sex	555 (73.5)	720 (73.5)	1.0
Body mass index, kg/m^2^	30.3 ± 6.0	29.9 ± 6.0	.07
Diabetes mellitus	300 (39.8)	415 (42.3)	.30
Hypertension	631 (83.7)	843 (86.0)	<.0001
Current cigarette smoker	179 (23.7)	171 (17.4)	.001
Cerebrovascular disease	54 (7.2)	81 (8.3)	.42
Peripheral vascular disease	76 (10.1)	97 (9.9)	.94
Prior MI	241 (32.0)	336 (34.3)	.33
Prior PCI	366 (48.5)	470 (48.0)	.85
Prior CABG	110 (14.6)	144 (14.7)	1.0
Indications for current PCI^a^			
Positive stress test and other	280 (37.1)	368 (37.6)	.88
Acute coronary syndrome	304 (40.3)	310 (31.6)	.0002
Chronic coronary syndrome	185 (24.5)	285 (29.1)	.04
STEMI	11 (1.5)	26 (2.7)	.10

Values are mean ± SD or n (%).

CABG, coronary artery bypass grafting; MI, myocardial infarction; PCI, percutaneous coronary intervention; STEMI, ST-segment elevation myocardial infarction.

a Multiple reasons could be recorded for any individual procedure. Other included patients undergoing PCI in preparation for noncardiac surgery, heart failure, and arrhythmia management.

Procedure characteristics are summarized in Table 2. Of note, radial access was used in approximately 60% of cases in both registries with 90% of procedures being single vessel PCI. The majority of procedures involved the treatment of a single lesion (75% in PRECISION and 73% in PRECISION GRX) with a median hospital length of stay of 1 day (1, 1). The use of contrast media was significantly higher in the PRECISION registry at a mean of 181.1 ± 90.8 mL compared to 118.3 ± 70.1 mL in PRECISION GRX (*P* < .0001). On average, 1.1 stents/lesion were implanted in PRECISION versus 1.2 stents/lesion in PRECISION GRX. Additional stents/lesions were placed in 11.3% of the PRECISION cohort and 18.5% of the PRECISION GRX cohort (5.5% and 4.4%, respectively due to geographic miss/insufficient lesion coverage). Postprocedure stenosis averaged 1.3% in both registries, a successful improvement from average pre-PCI stenosis of 85.2% in PRECISION and 86.3% in PRECISION GRX.

**Table 2:** Procedural characteristics of the study cohorts.

Characteristic	PRECISION (N = 754)	PRECISION GRX (N = 980)	*P* value
Access site			
Femoral	298 (39.5)	383 (39.1)	.80
Radial	452 (59.9)	594 (60.7)	
Other	4 (0.5)	3 (0.3)	
Preprocedure stenosis, %	85.2 ± 10.9 90.0 (80.0-95.0)	86.3 ± 9.9 90.0 (80.0-95.0)	.37
Postprocedure stenosis, %	1.3 ± 6.4 0 (0-0)	1.3 ± 8.3 0 (0-0)	.86
Lesion length, mm	17.4 ± 9.6 15.0 (10.0-22.0)	23 ± 17.9 18.0 (12.0-28.0)	<.0001
Stents implanted/lesion	1.1 ± 0.4 1.0 (1.0-1.0)	1.2 ± 0.6 1.0 (1.0-1.0)	.0022
Total fluoroscopy time, min	15.0 ± 8.4 13.0 (10.0-18.0)	17.8 ± 14.1 13.7 (8.0-22.9)	.10
Overall procedure time, min	61.9 ± 28.3 56.0 (43.0-74.0)	66.0 ± 36.8 47.0 (31.0-70.0)	.04
PCI procedure time, min	43.6 ± 23.1 38.0 (28.0-53.0)	54.3 ± 35.0 47.0 (31.0-70.0)	<.0001
Total contrast used for PCI, mL	181.1 ± 90.8 165.0 (120.0-225.0)	118.3 ± 70.1 100.0 (70.0-150.0)	<.0001

Values are n (%), mean ± SD, or median (IQR). Procedure time: “insertion of hemostatic sheath to removal of guide catheter”; PCI procedure time: “insertion of guide catheter to removal of guide catheter.”

PCI, percutaneous coronary intervention.

The average treated lesion length was 17.4 mm by visual assessment in PRECISION and 23.0 mm in PRECISION GRX, and the majority of treated lesions were type B2/C based on the American College of Cardiology/American Heart Association classification (Table 3). There were more target lesions treated in the left anterior descending artery in PRECISION compared to PRECISION GRX at 44.9% vs 36.8%, respectively, and fewer lesions in the right coronary artery at 27.3% in PRECISION and 33.9% in PRECISION GRX. A greater proportion of the lesions treated in the PRECISION GRX study were complex including a greater number of lesions with high angulation, moderate/severe calcification, at bifurcations, as well as more chronic total occlusions (CTO), and instances of in-stent restenosis. Notably, in PRECISION GRX, CTO were allowed to be crossed with a guidewire manually before being treated robotically (Table 3).

**Table 3:** Baseline angiographic characteristics of the study cohort.

Lesion characteristics	PRECISION (N = 950 lesions)	PRECISION GRX (N = 1233 lesions)	*P* value
Length (visual), mm	17.4 ± 9.6	23.0 ± 17.9	<0.0001
Reference vessel diameter, mm	3.0 ± 0.5	3.1 ± 0.6	.10
Lesion classification			<.0001
A	83 (8.7)	145 (11.8)	
B1	263 (27.7)	346 (28.1)	
B2	279 (29.4)	297 (24.1)	
C	325 (34.2)	445 (36.1)	
Target vessel			<.0001
Left main	12 (1.3)	36 (2.9)	
Left circumflex	252 (26.5)	325 (26.4)	
Left anterior descending	427 (44.9)	454 (36.8)	
Right coronary artery	259 (27.3)	418 (33.9)	
Vessel tortuosity			
None/mild	682 (71.8)	937 (76.0)	.03
Moderate	207 (21.8)	221 (17.9)	.02
Severe	61 (6.4)	75 (6.1)	.79
Lesion angulation			<.0001
≤45°	841 (88.5)	964 (78.2)	
>45°	109 (11.5)	269 (21.8)	
Lesion eccentricity			<.0001
Yes	543 (57.2)	538 (43.6)	
No	407 (42.8)	695 (56.4)	
Calcification			
None/mild	769 (80.9)	854 (69.3)	.0001
Moderate	134 (14.1)	245 (19.9)	.01
Heavy/severe	47 (5.0)	134 (10.9)	.007
Chronic total occlusion	25 (2.6)	110 (8.9)	<.0001
Bifurcation lesions	80 (8.4)	243 (19.7)	<.0001
In-stent restenosis lesion	59 (6.2)	159 (12.9)	.008
Long lesions (>20 mm)	200 (21.0)	273 (22.1)	.56

Values are n (%) or mean ± SD.

Clinical and technical success rates were similar between the 2 registries (Central Illustration). In the PRECISION registry, clinical success was 97.6% compared to 97.8% in PRECISION GRX. Technical success, calculated on a per-lesion basis and using the PRECISION GRX registry definition for the analysis, was 89.6% in PRECISION and 89.2% in PRECISION GRX. There was no significant difference between technical success rates using the technical definitions as predefined for each registry (Supplemental Figure S1). In PRECISION, technical success was defined as the proportion of procedures with clinical success in the absence of conversion to M-PCI, whereas in PRECISION GRX, it was defined as the successful completion of robotic-assisted PCI of the target lesion(s) without unplanned conversion to M-PCI owing to the inability of the guidewire or balloon stent/catheter to navigate the vessel anatomy or poor guide catheter support.

**Figure 2:**
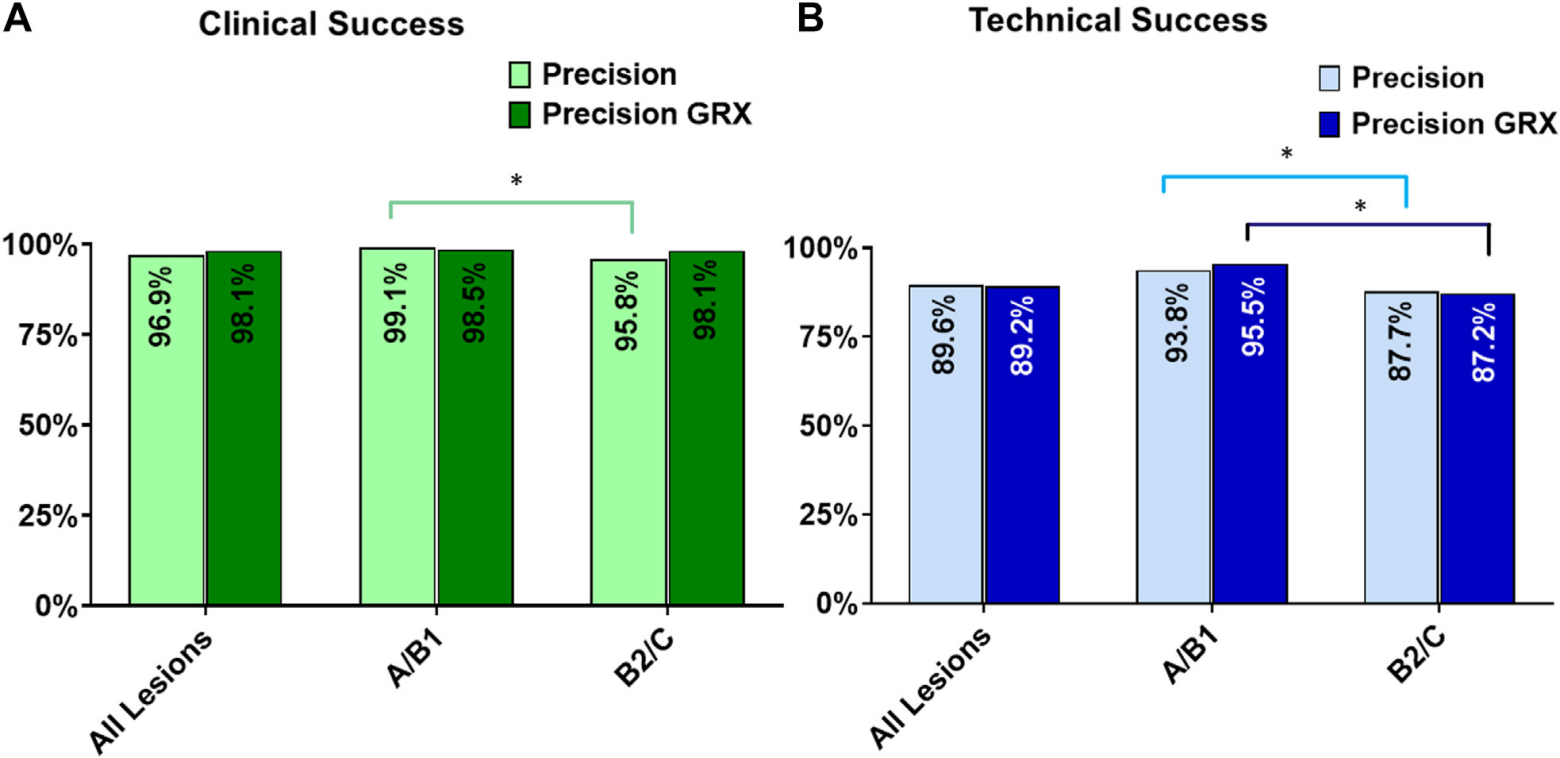
**Clinical and technical success: Stratification by lesion complexity in the PRECISION and PRECISION GRX registries.** Clinical success (by lesion) is defined as less than 30% residual stenosis (visual estimate) post percutaneous coronary intervention in the lesion(s) treated with the robotic system, without in-hospital major adverse cardiovascular event. Technical success (by lesion) utilizing the PRECISION GRX definition: successful completion of the robotic-assisted percutaneous coronary intervention without unplanned manual conversion. Notably, in PRECISION GRX, chronic total occlusions were predominantly treated as a hybrid approach with an initial manual crossing of the lesions. (**A**) Clinical success: PRECISION 96.9% (912/941) and PRECISION GRX 98.1% (1204/1227). Lesions A/B1 (PRECISION 99.1% [337/340] and PRECISION GRX 98.5% [324/329]) vs B2/C (PRECISION 95.8% [566/591] and PRECISION GRX 98.1% [709/723]). Clinical success was higher with type A/B1 lesions in PRECISION but comparable for both lesion groups in PRECISION GRX. (**B**) Technical success: PRECISION 89.6% (864/948) and PRECISION GRX 89.2% (1100/1233): Lesions A/B1 (PRECISION 93.8% [319/340] and PRECISION GRX 95.5% [315/330]) vs B2/C (PRECISION 87.7% [519/592] and PRECISION GRX 87.2% [635/728]). Technical success was higher in type A/B1 lesions in both PRECISION and PRECISION GRX. ∗*P* value < .05.

**Figure 3:**
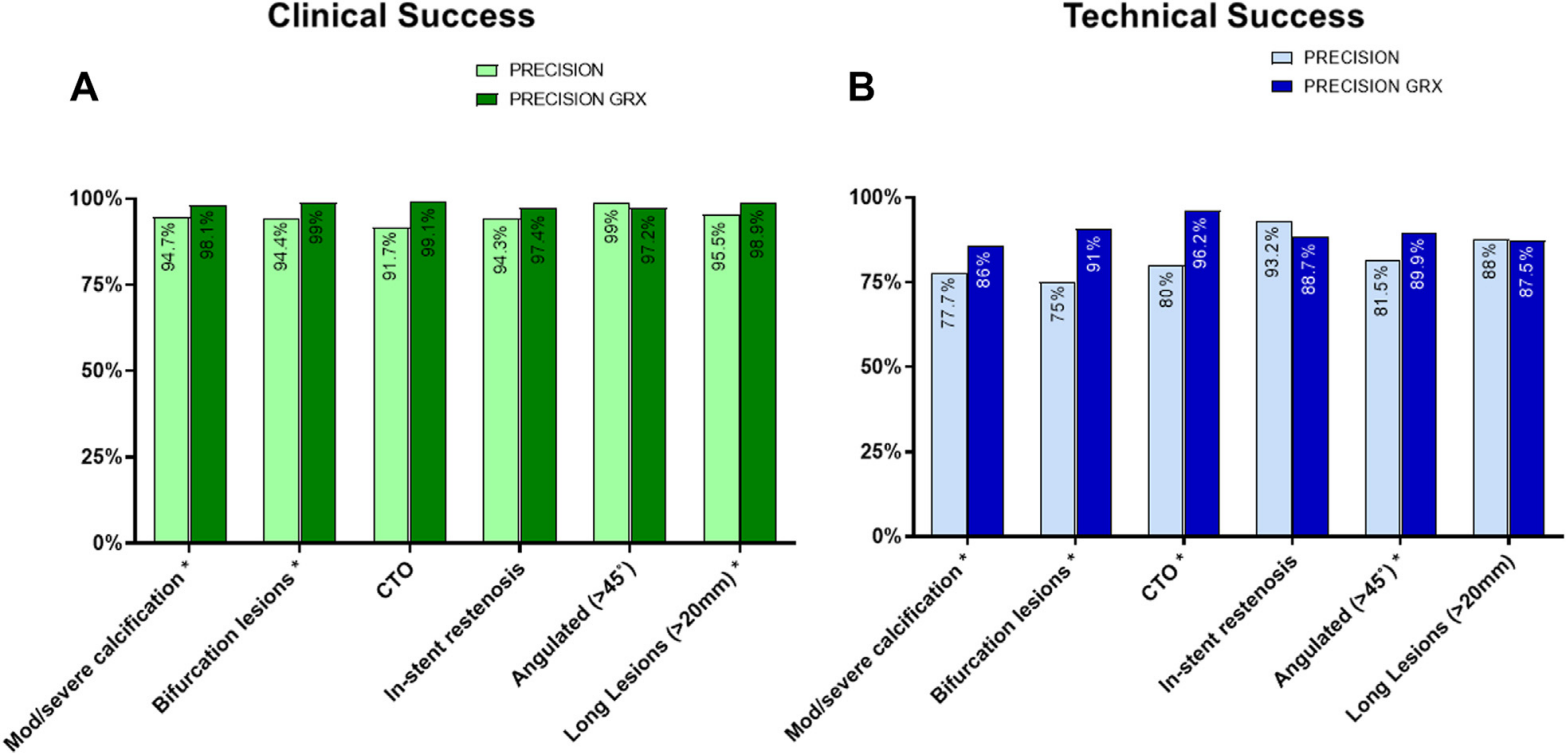
**Clinical and technical success: Complex lesion outcomes in the PRECISION and PRECISION GRX registries.** (**A**) Clinical success rates by lesion complexity. Higher clinical success was achieved in PRECISION GRX for: moderate/severe calcification lesions (PRECISION 94.7% [143/151] vs PRECISION GRX 98.1 [368/375]), bifurcation lesions (PRECISION 94.4% [68/72] vs PRECISION GRX 99% [205/207]), and long lesions (>20 mm) (PRECISION 95.5% [170/178] vs PRECISION GRX 98.9 [269/273]). (**B**) Technical success: by lesion complexity. Higher technical success was achieved in PRECISION GRX for: moderate/severe calcification lesions (PRECISION 77.7% [139/179] vs PRECISION GRX 86% [326/379]), bifurcation lesions (PRECISION 75% [60/80] vs PRECISION GRX 91% [192/211]), chronic total occlusions (CTO) (PRECISION 80% [20/25] vs PRECISION GRX 96.2% [102/106] and angulated (>45 °) lesions (PRECISION 81.5% [99/108] vs PRECISION GRX 89.9% [195/217]). Clinical success (by lesion) is defined as less than 30% residual stenosis (visual estimate) post percutaneous coronary intervention in the lesion(s) treated with the robotic system, without in-hospital major adverse cardiovascular event. Technical success (by lesion) utilizing the PRECISION GRX definition: successful completion of the robotic-assisted percutaneous coronary intervention without unplanned manual conversion. ∗ *P* value < .05.

Technical success rates were high across a range of lesion types. Technical success was achieved in 93.8% and 95.5% of type A/B1 lesions in PRECISION and PRECISION GRX, respectively. In more complex lesions (type B2/C), technical success rates were 87.7% in PRECISION and 87.2% in PRECISION GRX (Figure 2). For the combined PRECISION/PRECISION GRX data set, technical success was achieved in 89.4% of all lesions, 83.3% of lesions with moderate-to-severe calcification, 86.6% of bifurcation lesions, 93.1% of CTO, 89.9% of in-stent restenosis lesions, 87.1% of highly angulated lesions, and 87.7% of long lesions. Higher clinical success rates were observed in moderate/severe calcification lesions, bifurcation lesions, and long lesions with the second-generation CorPath GRX system. Similarly, higher technical success rates were observed with the second-generation CorPath GRX Robotic System in moderate/severe calcification lesions, bifurcation lesions, angulated lesions, and CTO (Figure 3).

In robotic procedures, conversion to M-PCI can be planned or unplanned. Planned conversion to M-PCI is typically temporary and is due to an inability to advance a device robotically. After the specific procedural step has been completed manually, a return to robotic control is common. Conversion to M-PCI occurred in 11.1% of treated lesions in PRECISION and 11.8% of lesions treated in PRECISION GRX (Supplemental Figure S1). The major reasons for manual conversion were unplanned, resulting in technical failure, and related to the inability to advance the interventional equipment or cross the lesion with the guidewire robotically (Table 4). Approximately 1% of procedures were converted manually for a device malfunction and none of those were associated with an adverse clinical event.

**Table 4:** Manual conversion and reasons (lesion level) of the study cohort.

Lesion characteristics Lesions	PRECISION (N = 950 lesions)	PRECISION GRX (N = 1233 lesions)
Manual conversion	105 (11.1)	145 (11.8)
Planned	6 (0.6)	12 (1.0)
Unplanned	99 (10.4)	133 (10.8)
Inability to advance gear	46 (4.8)	44 (3.6)
Inability to cross the lesion	29 (3.0)	46 (3.7)
Device malfunction	10 (1.1)	15 (1.2)
Clinical condition deterioration	9 (0.9)	9 (0.7)
Other	25 (2.6)	32 (2.6)

VAlues are n (%). Multiple reasons could be recorded for any individual procedure.

In-hospital MACE rates were low both in PRECISION and PRECISION GRX. In PRECISION, MACE events included 5 non–Q-wave MI and 1 ischemic stroke. MACE for PRECISION GRX were 1 ischemic stroke and 1 repeat PCI on a nontarget vessel. There were no deaths in either registry. Other adverse events were typical of those reported in PCI, including access site complications, vessel dissection, and acute kidney injury. No serious adverse events related to the CorPath robotic systems were observed in either registry.

## Discussion

This is the first multicenter comprehensive report addressing the safety and efficacy of R-PCI in clinical practice. It demonstrates that R-PCI with both the first-generation and second-generation robotic platforms is safe with very low adverse events and none related to the robotic platform. High clinical success rates were observed in a broad representative patient population as treated by multiple operators in clinical practice. The lesions treated in the PRECISION and PRECISION GRX studies were more complex than reported in previous studies,^3^, ^4^, ^5^^,^^7^^,^^8^ and high technical success rates were observed with both generation platforms. Furthermore, lesions treated with the second-generation platform in PRECISION GRX were more complex with a higher proportion of moderate and severely calcified lesions, bifurcation lesions, CTO, and in-stent restenosis lesions. The ability to control the guide catheter robotically and additional newer software features with the second-generation robotic system might have helped achieve higher technical success in these more complex lesions.

Robotic PCI was developed to help address the occupational hazards associated with the practice of interventional cardiology including radiation exposure and orthopedic injuries. With the multiple advances in the technical aspects of interventional cardiovascular medicine, increasingly complex coronary lesions are being treated percutaneously. The early feasibility study with R-PCI^3^ enrolled relatively straightforward lesions and the question remained regarding the utility of R-PCI in contemporary clinical practice. Although previous studies have shown that complex lesions can be treated robotically,^4^^,^^5^^,^^8^ the major limitations of those studies have been the single center and/or single operator experience. The PRECISION and PRECISION GRX studies are the first set of studies to systematically address the safety and efficacy of R-PCI in a broad range of patients, lesions, and clinical presentations, with both experienced and novice operators. There were no robotic platform–related complications resulting in an adverse event in any patient in both registries. This is of paramount importance for any new technology and provides reassurance that the interventional operator can be in the interventional cockpit or even in the control room, away from the catheterization table managing the robot. Nevertheless, a tableside team is still required to assist in device exchanges and rapid manual assistance as required.

This large multicenter multioperator experience shows that R-PCI is safe and can be used to treat a broad range of coronary lesions in patients presenting for PCI for elective, stable angina, and acute coronary syndrome, including ST-elevation myocardial infarction indications. The clinical success rates observed with both the first- and second-generation platforms are representative of the high success of PCI in contemporary clinical practice. The 60% radial access in both registries is also demonstrative of the change in clinical practice in the United States during the duration of the studies and shows that R-PCI can be performed regardless of the arterial access site.

The lesions treated in both studies were significantly more complex than previous reports. The majority of lesions treated were type B2/C but the technical success rates in the more complex lesions were lower than those achieved in type A/B1 lesions. In the more complex lesions, multiple reasons for manual assistance or conversion were observed. The majority of these manual conversions in the first-generation robotic system (CorPath 200) were related to the inability to control the guide catheter robotically and, as a result, with the advancement of the balloon catheter or stent delivery system, guide disengagement required transient manual assistance. This issue was addressed by the second-generation robotic system and this could have been 1 potential reason that more complex lesions were treated robotically in PRECISION GRX. Additional reasons for manual assistance or conversion included the inability to address bifurcation lesions when side-branch stenting was required in a provisional approach as the robotic drive only enables a single delivery system; calcified lesions which did not yield with a balloon requiring bailout atherectomy; intravascular coronary imaging; and lesions requiring guide catheter extension placement manually.

Although procedure and fluoroscopy times were longer in the PRECISION GRX study, contrast utilization was lower. It is possible that for more complex lesions, although the procedure time was prolonged, operators used less contrast during the R-PCI procedure due to additional software features facilitating easier and automated guidewire advancement. Furthermore, with the second-generation platform, a high-resolution imaging screen was positioned in the robotic cockpit which might have also enabled superior visualization of the relevant coronary anatomy and resulted in lower contrast use. Notably, overall procedure time with both platforms was longer than nonrobotic PCI procedures. This is consistent with a previous report of R-PCI prolonging an average PCI procedure by 8 minutes as compared to M-PCI.^5^ However, in the absence of an M-PCI control group in the current registries, limited interpretation of these procedural times can be made.

These studies provide the foundation to further investigate remote R-PCI (tele-stenting).^9^^,^^10^ Future iterations of the system require the development of a second robotic drive to enable bifurcation stenting and advancement of guide extension catheters. As the use of intravascular imaging is increasing, it will likely also be incorporated into the robotic system. Additional development for an over-the-wire platform would assist in robotic atherectomy, thrombectomy, and CTO revascularization entirely robotically.

There are certain limitations of both registry studies. The decision to do R-PCI vs M-PCI was at the operator’s discretion and not protocol-driven. It is possible that specific complex procedures, such as those with planned atherectomy, intravascular imaging, or CTO lesions were excluded by the operators. However, the procedure characterization shows that a good proportion of the procedures were complex and planned as hybrid manual-robotic (eg, CTO). Minor adverse clinical outcomes might have been underreported as is common in most registry studies. However, the data were audited against source information and this was unlikely to affect the primary reported end points. The outcomes regarding robotic CTO revascularization are confounded in the PRECISION GRX registry as manual crossing of the chronically occluded artery was performed prior to connecting the robotic drive. Therefore, success for this subgroup of complex coronary lesions was not entirely dependent on the robotic system. Data regarding resource utilization including contrast utilization, fluoroscopy exposure, and patient radiation exposure were evaluated in an exploratory manner between the first- and second-generation robotic systems but without a manual control group, and could not provide any definitive interpretations.

In conclusion, this comprehensive analysis of the PRECISION and PRECISION GRX studies demonstrates that R-PCI with both the CorPath 200 and CorPath GRX systems is safe, and results in high clinical and technical success. In more complex lesions, the second-generation CorPath GRX system results in higher technical success likely due to additional features including guide catheter support. These studies provide data supporting the use of R-PCI in routine clinical practice.

## Figures and Tables

**Supplementary Figure S1:**